# Mutations in *TSPAN12* gene causing familial exudative vitreoretinopathy

**DOI:** 10.1186/s40246-024-00589-6

**Published:** 2024-03-01

**Authors:** Yuqiao Ju, Tianhui Chen, Lu Ruan, Ye Zhao, Qing Chang, Xin Huang

**Affiliations:** 1 Department of Ophthalmology and Vision Science, Eye and ENT Hospital of Fudan University, 83 Fenyang Rd, Shanghai, 200031 China; 2 Key Laboratory of Myopia of State Health Ministry, and Key Laboratory of Visual Impairment and Restoration of Shanghai, Shanghai, China

**Keywords:** Missense mutation, PTC, NMD, TSPAN12, Familial exudative vitreoretinopathy, UPF1

## Abstract

**Background:**

To report newly found *TSPAN12* mutations with a unique form of familial exudative vitreoretinopathy (FEVR) and find out the possible mechanism of a repeated novel intronic variant in *TSPAN12* led to FEVR.

**Results:**

Nine *TSPAN12* mutations with a unique form of FEVR were detected by panel-based NGS. MINI-Gene assay showed two splicing modes of mRNA that process two different bands A and B, and mutant-type shows replacement with the splicing mode of Exon11 hopping. Construction of wild-type and mutant *TSPAN12* vector showed the appearance of premature termination codons (PTC). In vitro expression detection showed significant down-regulated expression level of *TSPAN12* mRNAs and proteins in cells transfected with mutant vectors compared with in wild-type group. On the contrary, translation inhibitor CHX and small interfering RNA of UPF1 (si-UPF1) significantly increased mRNA or protein expression of *TSPAN12* in cells transfected with the mutant vectors.

**Conclusions:**

Nine mutations in *TSPAN12* gene are reported in 9 FEVR patients with a unique series of ocular abnormalities. The three novel *TSPAN12* mutations trigger NMD would cause the decrease of TSPAN12 proteins that participate in biosynthesis and assembly of microfibers, which might lead to FEVR, and suggest that intronic sequence analysis might be a vital tool for genetic counseling and prenatal diagnoses.

**Supplementary Information:**

The online version contains supplementary material available at 10.1186/s40246-024-00589-6.

## Background

Familial exudative vitreoretinopathy (FEVR, OMIM 133780) is hereditary angiogenesis of the retina whose characteristic is peripheral avascularity in the retina, described by Schepens and Criswick firstly in 1969 [1]. The retinal vascular abnormalities in FEVR might lead to incomplete and aberrant vascularization resulting in retinal fold, detachments, retinal neovascularization, fibrosis, folds, subretinal exudation, vitreous hemorrhage, macular ectopia, and eventually causing to total blindness. In China, FEVR was found in 217 infants (1.19% of all abnormalities) among 199,851 newborns reported according to a multicenter research.

FEVR can be genetically heterogeneous and inherited autosomal dominantly, autosomal recessively, or X-linked. Ten genes, including *TSPAN12, FZD4, LRP5, KIF11*, have been identified to the cause of FEVR [2–4]*. TSPAN12* mutations have been reported in FEVRs with an autosomal dominant inheritance, which belongs to the phylogenetically ancient family of tetraspanins. However, a thorough understanding of the molecular mechanisms underlying TSPAN12 activity remains unclear.

TSPAN12 functions as a signaling platform within the plasma membrane whose relations to the Norrin/Frizzled4 signalling pathway have been demonstrated [5]. Norrin/Frizzled4 signaling was identified as a classic passthway causing the retinal vascular disease FEVR. According to Poulter et al., it was examined that TSPAN12 is crucial for Norrin/Frizzled4 signaling pathway, which is responsible for the development of avascular peripheral retinas and various other vitreoretinopathies. Junge et al. found that TSPAN12 plays a specific role in controlling Norrin/b-catenin signaling, rather than Wnt/b-catenin signaling, through its influence on FZD4 multimerization [6].

Herein, nine pathogenic mutations were screened for on 117 unrelated FEVR patients and afterwards the ocular manifestations were examined and analyzed in accordance with the mutation spectrum.

## Methods

### Study subjects and clinical assessments

This study focused on nine different FEVR patients with *TSPAN12* mutation and their relatives who attended clinics and were diagnosed at the Eye and ENT Hospital of Fudan University, Shanghai, China. Informed consent has been obtained from all participants and written consent has been provided to us. All experiments were conducted as per the Helsinki Declaration tenets. This study was conducted in accordance with the approval fron the Ethical Oversight Committee of Eye and ENT Hospital of Fudan University. FEVR was evaluated according to the previously described diagnostic criteria [7]. In brief, the diagnosis of FEVR can be established by using fundus photography, slit-lamp biomicroscopy, type-B ultrasonic imaging, or fundus fluorescein angiography (FFA) by ophthalmologists according to the following criteria: (1) full term birth and no history of oxygen inhalation; (2) retinal avascular areas; and (3) retinal neovascularization or vascular leakage.

### Whole exome sequencing and data analysis

The procedure of whole exome sequencing was done in Shanghai We-Health Biomedical Technology Co., Ltd. Genomic DNA samples obtained from individuals with FEVR were isolated from peripheral leukocytes utilizing a commercially available extraction kit (TIANGEN, China). The quality/quantity of DNA was evaluated utilizing Onedrop OD1000 spectrophotometer and by agarose gel electrophoresis. Exome capture was conducted with xGen Exome Research Panel v1.0 (Integrated DNA Technologies, Inc., Iowa, United States) and 150 base pair paired end sequencing was executed using the Illumina HiSeq4000 platform (San Diego, CA).The unprocessed reads were aligned using the Burrows–Wheeler Aligner (BWA) and SAMtools by the sequencing company. Then after eliminating duplicates from sorted alignment using Picard, variants were called using the Genome Analysis Toolkit (GATK v3.70) pipeline.

### Variant validation

The suspected causal gene variants thereby discovered were demonstrated through Sanger sequencing. Genomic DNA was isolated from peripheral leukocytes ultilizing TIANamp Blood DNA Kit (TiangenBiotech, Beijing, China). Primers were designed using the Primer3Plus software (http://www.primer3plus.com/cgibin/dev/primer3 plus.cgi). PCR amplification was peformed by pre-denaturation at 95 °C for 5 min, and subsequently 35 cycles of 95 °C for 30 s, 60 °C for 30 s and 72 °C for 1 min, with an extension at 72 °C for 5 min in the end. The products of PCR were then examined by 2% agarose gelelectrophoresis. Then the PCR products were sequenced directionally on an ABI3730xl DNA Analyzer (AppliedBiosystems, FosterCity, California, USA).

### Construction of MINI-gene and transcription analysis

#### TSPAN12: c.612+1G>A

Based on the mutation information, mutant and wild-type plasmids were constructed. The cloning vehicle was pMini-CopGFP, with a cloning site of BamHI/XhoI. Three pairs of nested primers TSPAN12-612-AF/TSPAN12-612-AR, TSPAN12-612-BF/TSPAN12-612-BR and TSPAN12-612-CF/TSPAN12-612-CR were designed to amplify the genomic DNA of FEVR 48 (TSPAN12: c.612+1G>A) (intron 6 and 7 were too large and the target fragment was amplified in three segments, and the products were named TSPAN12-612A, TSPAN12-612B and TSPAN12-612C, respectively), and mutant and wild-type target fragments were acquired and by digestion and recombination reactions inserted into the cloning vector. The primer information is shown in Additional file 1.

#### TSPAN12: c.149+2T>C

Two pairs of nested primers TSPAN12-149-AF/TSPAN12-149-AR and TSPAN12-149-BF/TSPAN12-149-BR were designed to amplify the genomic DNA of FEVR 91 (intron 2 were too large and the target fragment was amplified in two segments, and the products were named TSPAN12-149A and TSPAN12-149B). The rest of the steps are as described previously. The primer information is shown in Additional file 1.

#### TSPAN12: c.360G>Ac

Two pairs of nested primers TSPAN12-360-AF/TSPAN12-360-AR, TSPAN12-360-BF/TSPAN12-360-BR and TSPAN12-360-CF/TSPAN12-360-CR were designed to amplify the genomic DNA of FEVR 104 (intron 4 and 5 were too large and the target fragment was amplified in two segments, and the products were named TSPAN12-360A, TSPAN12-360B and TSPAN12-360C). The rest of the steps are as described previously. The primer information is shown in Additional file 1.

Followed by incubation at 4 °C overnight, the recombinant vector was transformed into *E. coli* competent cells *E.coli* DH5ɑ. Culture 5–15 ml of bacterial broth, after 12 h of incubation, single clones were picked by PCR amplification and Sanger sequencing to identify if the target fragments of TSPAN12 gene was accurately inserted into the vectors. The correctly recombinant mutant minigene plasmids and wild-type minigene plasmids were picked out. TIANprep Mini Plasmid Kit (DP103-03) was used for vectors extraction according to the guidence of instruction book. The identified primers were shown in Additional file 1.

### Transfection of recombinant vector with TSPAN12

293T cells were cultured using DMEM + 10% FBS. Wt and mut recombinant vectors were transiently transfected into 293T by Liposomal Transfection Reagent (40802ES03) according to the guidance of the instruction. Samples were collected after 48 h of incubation.

### RT-PCR and Sanger sequence

The transfected cells were collected to extract RNA reverse transcribed cDNA. For RT-PCR amplification, primers were designed, while gel electrophoresis was performed. PCR amplification products were subjected to Sanger sequencing. The RT-PCR primer sequences are shown in Additional file 1.

## Results

### Clinical data

**Fig. 1:**
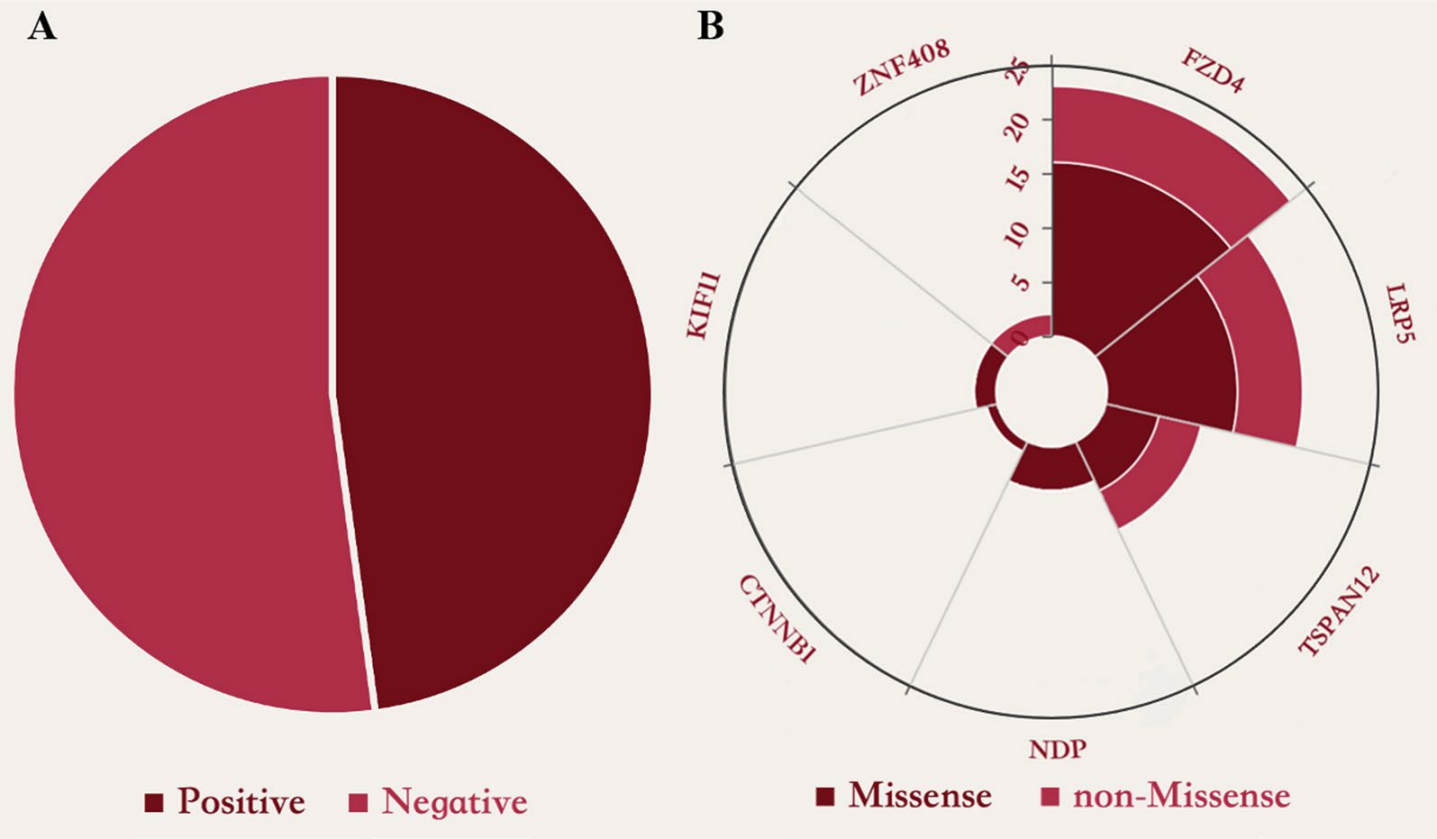
**A** Distribution of gene mutations associated with FEVR in 117 probands, with positive results accounting for 47.87% of cases. **B** Proportions of mutations of all positive cases classified as missense mutations and non-missense mutation

Next-generation sequencing (NGS) of the DNA samples from each patient can determine the genetic cause of FEVR. A total of 117 subjects received a panel-based NGS test in Fudan University Eye and ENT Hospital between 2015 Jan and 2022 Dec. The demographic characteristics of the overall patients are compiled below within Table 1. Among the variants recognized in these study participants, there were approximately 47.87% (56 of 117) of variants were found in 7 genes (FZD4, LRP5, TSPAN12, NDP, CTNNB1, ZNF408, and KIF11) (Table 2). 9 probands were identified as carrying TPSAN12 mutation, with a mutation detection rate of 15.25%. The proportion of missense mutations in positive mutated genes is shown in Table 2 and Fig. 1B. The highest non-missense mutation proportion was TSPAN12, reaching 44.44%.

**Table 1:** Demographic characteristics of the overall cohort

NGS results	Number of cases	Proportion (%)
Positive	56	47.87
Negative	61	52.13

**Table 2:** Overall spectrum of identified variants and proportions of mutation types

Gene	Number	Proportion (%)	Missense	Non-Missense	Propotion of non-Missense (%)
FZD4	23	38.98	16	7	30.43
LRP5	18	30.51	12	6	33.33
TSPAN12	9	15.25	5	4	44.44
NDP	4	6.78	4	0	0.00
CTNNB1	1	1.69	1	0	0.00
ZNF408	2	3.39	2	0	0.00
KIF11	2	3.39	0	2	100.00

**Fig. 2:**
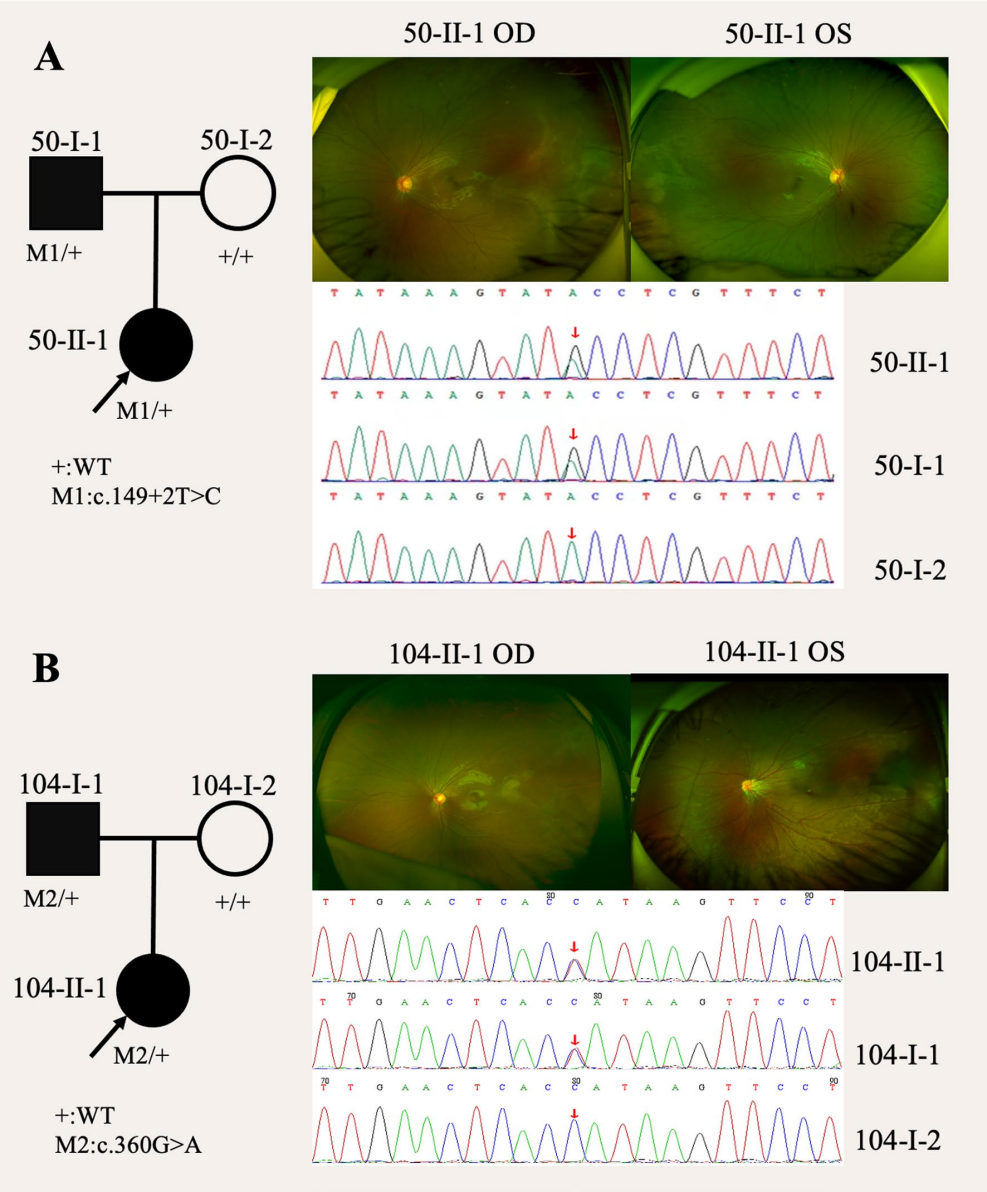
Identification of *TSPAN12* mutations in FEVR patients. **A** Pedigrees of patients FEVR50. The black arrow shows the proband in the family. Ocular phenotypes associated with patients with *TSPAN12* mutation. Sequence verification and segregation analysis of *TSPAN12* mutations. The red boxes show the variants identified in the probands. **B** Pedigrees of patients FEVR104. The black arrow shows the proband in the family. Ocular phenotypes associated with patients with *TSPAN12* mutation. Sequence verification and segregation analysis of *TSPAN12* mutations. The red boxes show the variants identified in the probands

The baseline characteristics of the 9 probands with TPSAN12 mutation are summarized in Table 3. In this cohort, 6 were female and 3 were male. The age of these patients ranging from 1 to 54 years. Overall, 7 patients were found to have the positive family history. Fathers of five of the 9 patients had FEVR histories and the mothers of two had histories of FEVR patients. The fathers of FEVR 50 and FEVR 104 carried the same genetic variant. Cosegregation analysis of FEVR50 and FEVR104 demonstrated a trans pattern and confirmed the autosomal dominant inheritance (Fig. 2). All 9 patients underwent graded assessment for FEVR stage, measurement of BCVA, and examination of associated pathology. FEVR 29 showed FEVR stage 4 in his left eye, with his BCVA not being detected, and the FEVR stage of the remaining 17 eyes were normal (stage 1). FEVR 29 had retinal detachment in her left eye and accepted the scleral bucking surgery. A total of 8 eyes in 4 patients developed drag-disc.

**Table 3:** Baseline characteristics of FEVR patients with TSPAN12 mutations

No.	Age of onset	Sex	Family history	Eye	FEVR Stage	BCVA	Associated pathology		
							Drag-disc	Retinal Detachment	Treatment
FEVR 13	54	F	N/A	OD	1	0.6			
				OS	1	1			
FEVR 29	11	F	Father	OD	1	N/A			
				OS	4A	N/A		√	Scleral bucking surgery
FEVR 40	15	F	N/A	OD	1	0.6			
				OS	1	1			
FEVR 45	8	M	Father	OD	1	0.05	√		
				OS	1	1	√		
FEVR 48	1	F	Mother	OD	1	N/A			
				OS	1	N/A			
FEVR 50	6	M	Father	OD	1	0.9			
				OS	1	0.9			
FEVR 104	9	F	Father	OD	1	0.6	√		
				OS	1	0.05	√		
FEVR 106	6	F	Mother	OD	1	0.02	√		
				OS	1	0.8			
FEVR 113	9	M	Father	OD	1	0.3	√		
				OS	1	1	√		

*F* female, *M* male, *OD* right eye, *OS* left eye, *BCVA* best corrected visual acuity

### Patients with TSPAN12 variants

The genetic spectrum are as follows (Table 4). A total of 9 gene variants were found in the TPSAN12, of which 6 variants, c.402G>T, p.R134S; c.287A>G, p.Y96C; c.232G>A, p.G78R; c.149+2T>C, -; c.360G>A, p.M120I; c.449G>A, p.W150X, were novel, and 3 variants, c.352G>T, p.E118X; c.612+1G>A, -; c.146C>T, p.T49M, has been previously reported. Overall, the majority of probands carried missense mutations (55.56%). 5 were missense mutations, 2 were nonsense and 2 were splice site mutations. Four of the 9 TSPAN12 mutations were categorized as “pathogenic” or “likely pathogenic”, while the remaining missense mutations were considered variants of uncertain significance (VUS) in accordance with the guidelines of ACMG. The scores of Mutation Taster, Splice AI and SPIDEX predicted that the variants can negatively impact the protein's functionality. FEVR 13 was detected to have missense mutation c.402G>T (p.R134S), while Sample 40 had missense mutation c.287A>G (p.Y96C). Sample 45 was examined to have a missense variant c.232G>A (p.G78R). Sample 104 was identified to have a missense mutation c.360G>A (c.286_360del), which has been proved to induce exon 5 skipping or not. Sample 113 was also examined to have a missense variant c.146C>T (p.T49M) of TPSAN12. In addition, Sample 29 and Sample 106 had nonsense mutations c.352G>T (p.E118X) and c.449G>A (p.W150X), respectively. Sample 48 and Sample 50 had splice site mutations c.612+11G>A (c.469_612del in exon 8) and c.149+2T>C (c.67_149del in exon 3), respectively.

**Table 4:** The Overall Spectrum of TSPAN12 Variants Found in This Study

Sample	Gene	Nucleotide changes	Protein changes	Reference	Effect	Mutation	Splice	SPIDEX	gnomAD	ACMG	Heredity
						Taster	AI				
FEVR 13	TSPAN12	c.402G>T	p.R134S	Novel	Missense	DC	–	0.2613	–	VUS	AD
FEVR 29	TSPAN12	c.352G>T	p.E118X	Reported	Nonsense	DC	–	− 52.5689	–	P	AD
FEVR 40	TSPAN12	c.287A>G	p.Y96C	Novel	Missense	DC	F(0.06)	− 0.1202	–	VUS	AD
FEVR 45	TSPAN12	c.232G>A	p.G78R	Novel	Missense	DC	–	0.079	−	VUS	AD
FEVR 48	TSPAN12	c.612+1G>A	–	Reported	Splice variation	DC	T(1)	− 30.3463	–	P	AD
FEVR 50	TSPAN12	c.149+2T>C	–	Novel	Splice variation	DC	T(0.91)	− 8.9528	–	LP	AD
FEVR 104	TSPAN12	c.360G>A	p.M120I	Novel	Missense	DC	T(0.74)	− 0.6573	–	VUS	AD
FEVR 106	TSPAN12	c.449G>A	p.W150X	Novel	Nonsense	DC	–	− 48.1427	–	LP	AD
FEVR 113	TSPAN12	c.146C>T	p.T49M	Reported	Missense	DC	F(0.03)	− 5.0843	0.00000399	LP	AD

*DC* disease causing, *LP* Likely pathogenic, *P* Pathogenic, *VUS* variants of uncertain significance, *AD* autosomal dominant

**Fig. 3:**
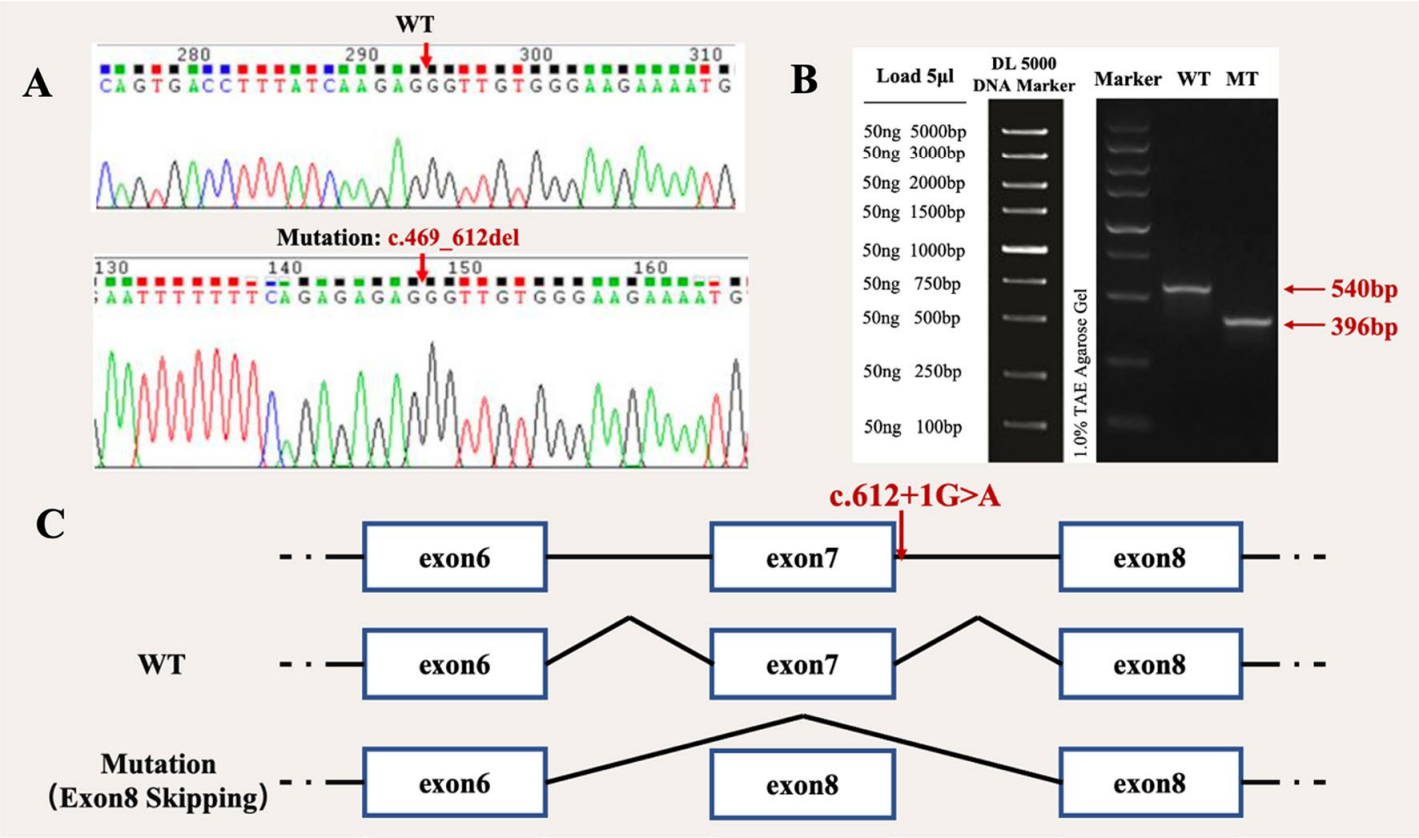
Vector construction and analysis of *TSPAN12* expression. **A** Sequencing results showed successful introduction of mutation c.496_612del (-) into recombinant plasmids. **B** Expression of mut and wt TSPAN12 proteins was examined by Western blot, and the result showed that the protein translated by the cells transfected with the mut vector was significantly lower than proteins which were translated by the cells transfected with the WT plasmid. **C** The mutation c.612+1G>A cause exon 7 skipping

**Fig. 4:**
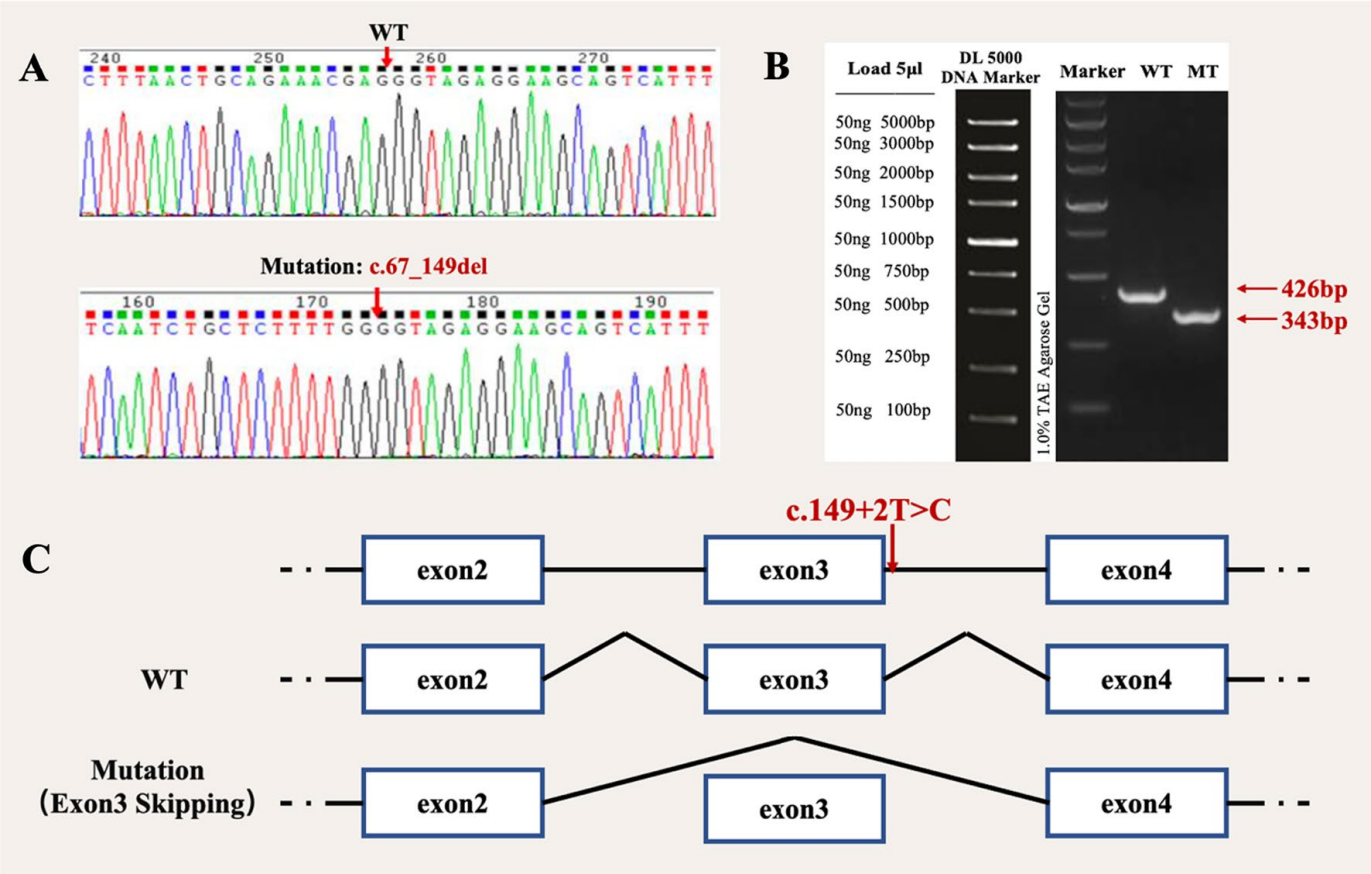
Vector construction and analysis of *TSPAN12* expression. **A** Sequencing results showed successful introduction of mutation c.67_149del (-) into recombinant plasmids. **B** Expression of mut and wt TSPAN12 proteins was examined by Western blot, and the result showed that the protein translated by the cells transfected with the mut vector was significantly lower than proteins which were translated by the cells transfected with the WT plasmid. **C** The mutation c.149+2T>C cause exon 3 skipping

**Fig. 5:**
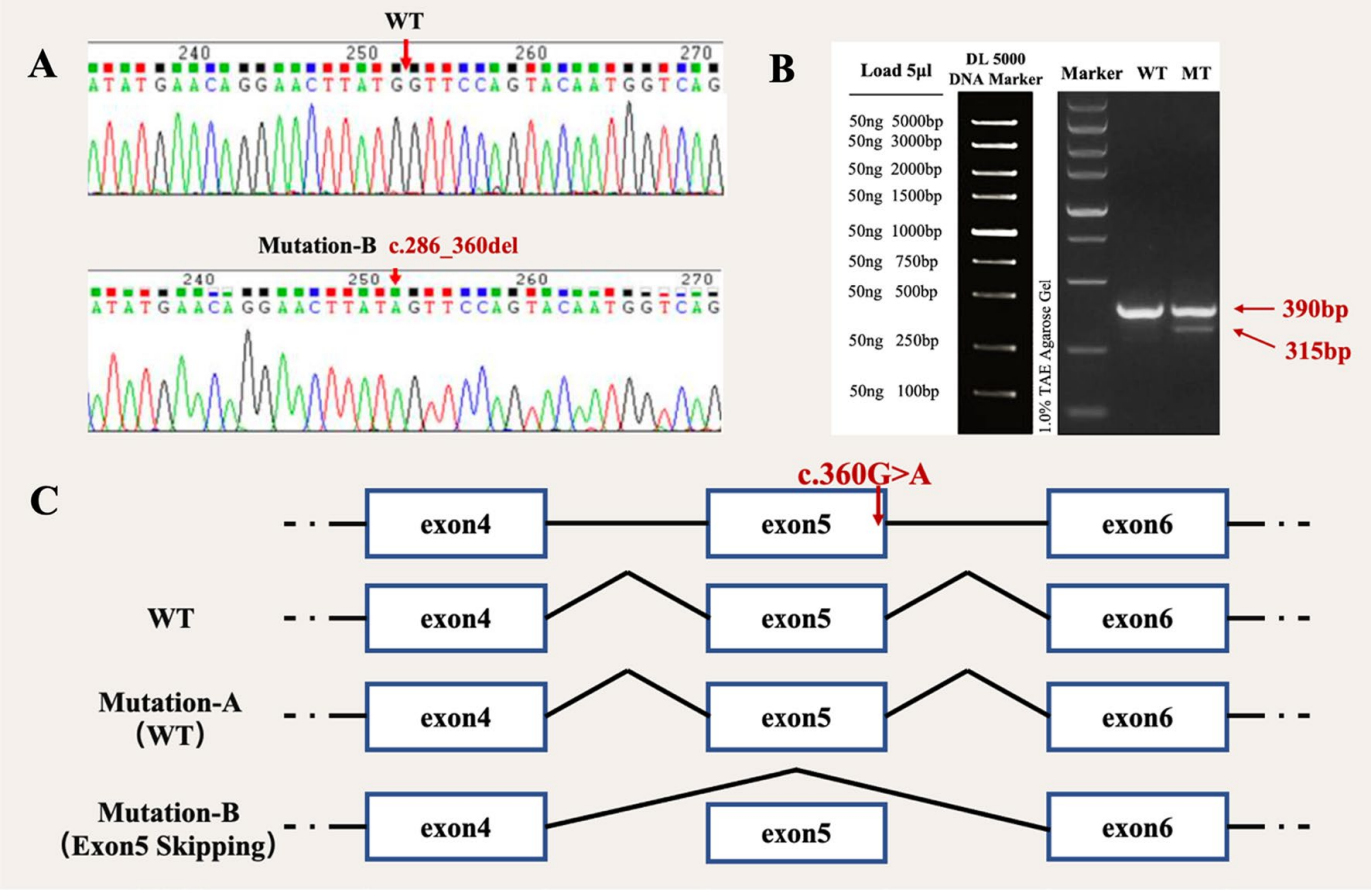
Vector construction and analysis of *TSPAN12* expression. **A** Sequencing results showed successful introduction of mutation c.286_360del (p.M120I) into recombinant plasmids. **B** Expression of mut and wt TSPAN12 proteins was examined by Western blot, and the result showed that the protein translated by the cells transfected with the mut vector was significantly lower than proteins which were translated by the cells transfected with the WT plasmid. **C** The mutation c.360G>A cause exon 5 skipping or not

In order to explore the possibility of a novel splicing mode during gene transcription resulting from the *TPSPAN12* mutation, transfection experiments were conducted using wild-type and mutant *pcMINI-TPSPAN12* vectors respectively in 293T and HeLa cells. PCR and sequencing of recombinant plasmids showed the successful incorporation of both wild-type and mutant minigenes into their respective vectors (Figs. 3, 4 and 5). The extraction of total RNA was performed for RT-PCR. Results shows that in 293T cells and Hela cells. The splicing mode of FEVR 48 mutant-type band were reduced from 540 to 396 bp, causing exon 7 skipping (Fig. 3). The splicing mode of FEVR 50 mutant-type band were reduced from 426 to 343 bp, causing to exon 3 skipping (Fig. 4). There were one bands of the c.360G>A of FEVR 104, of which band was 390 bp in wild-type. The mutant-type exhibited a dual band pattern, designated as A and B correspondingly, of which band A was 390 bp in expected size and band B was smaller (Fig. 5B). These two bands were sent for sequencing and results revealed that band A of wild-type was the normal splicing band, whose splicing mode was exon 4—exon 5—exon 6.

### The predicted 3D protein structures of TPSAN12 variants

The protein TPSAN12 was subjected to 3-Dimensional structure prediction using Swiss-Pdb Viewer 4.1, revealing that the variants could potentially affect the functionality of the proteins by disrupting hydrogen bonding, or making skipping. The absence of an appropriate model in the SWISS-MODEL database hindered the construction of TPSAN12 protein structure, considering its splice variation.

**Fig. 6:**
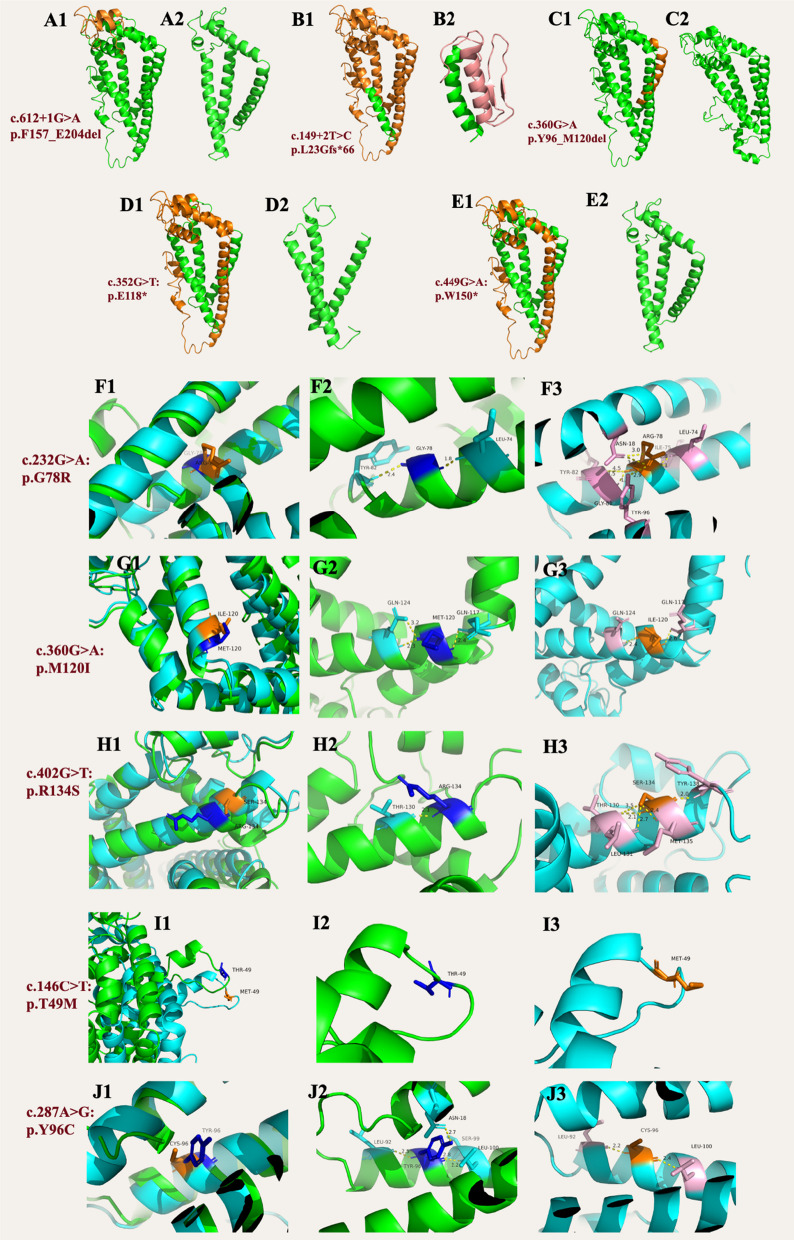
The predicted effect of *TSPAN12* variants in 3D protein structures. **A1** The structure of wild type TPSAN12, the missing amino acids F157-E204 due to mutation c.612+1G>A is shown in orange. **A2** The structure of TPSAN12 with mutation c.612+1G>A. **B1** The structure of wild type TPSAN12, the changed amino acids L23–L305 due to mutation c.149+2T>C is shown in orange. **B2** The structure of TPSAN12 with mutation c.149+2T>C. **C1** The structure of wild type TPSAN12, the missing amino acids Y96-M120 due to mutation c.360G>A is shown in orange. **C2** The structure of TPSAN12 with mutation c.360G>A. **D1** The structure of wild type TPSAN12, the missing amino acids E118–L305 due to mutation c.352G>T is shown in orange. **D2** The structure of TPSAN12 with mutation c.352G>T. **E1** The structure of wild type TPSAN12, the missing amino acids W150–L305 due to mutation c.449G>A is shown in orange. **E2** The structure of TPSAN12 with mutation c.449G>A. **F1** Superposition before and after mutation, with green representing the wild type and blue representing the mutant type p.G78R. **F2** The structure of GKY-78 and its surrounding residues. **F3** The structure of the mutation ARG-78 and its surrounding residues. **G1** Superposition before and after mutation, with green representing the wild type and blue representing the mutant type p.M120I. **G2** The structure of MET-120 and its surrounding residues. **G3** The structure of the mutation ILE-120 and its surrounding residues. **H1** Superposition before and after mutation, with green representing the wild type and blue representing the mutant type p.R134S. **H2** The structure of ARG-134 and its surrounding residues. **H3** The structure of the mutation SER-134 and its surrounding residues. **I1** Superposition before and after mutation, with green representing the wild type and blue representing the mutant type p.T49M. **I2** The structure of THR-49 and its surrounding residues. **I3** The structure of the mutation MET-49 and its surrounding residues. **J1** Superposition before and after mutation, with green representing the wild type and blue representing the mutant type p.Y96C. **J2** The structure of TYR-96 and its surrounding residues. **J3** The structure of the mutation CYS-96 and its surrounding residues

The predicted protein structures of TSPAN12 are used to better explain the potential pathogenic effects and protein function of TSPAN12 mutation variants. Mutations of c.612+1G>A, c.149+2T>C and c.360G>A could affect mRNA splicing which cause exon skipping. Nonsense mutations c.352G>T and c.449G>A could cause premature termination of transcription. Mutation of c.232G>A: p.G78R could cause non-polar amino acids becoming polar amino acids, which affects the protein structure: wild-type GKY-78 formed hydrogen bonding with TYR-82 at a spacing of 2.4 Å and with LEU-74 at a spacing of 1.8 Å; mutant ARG-78 formed hydrogen bonds with several surrounding amino acids including TYR-82, LEU-74, TRY-96, and GLY-81. Mutation c.360G>A could produce two forms of mRNA splicing, of which one mutation affecting mRNA splicing shown above, another mutation p.M120I could cause polar amino acid becoming non-polar amino acid, which affects the TSPAN12 protein structure: wild-type MET-120 forms hydrogen bonds with spacing of 2.3 Å and 3.2 Å with GLN-124 and 2.4 Å with GLN-117; mutant ILE-120 forms hydrogen bonds with spacing of 2.4 Å with GLN-124 and 2.6 Å with GLN-117. Mutation of c.402G>T: p.R134S could cause basic amino acid changed to neutral amino acid, which affects the TSPAN12 protein structure: wild-type ARG-134 formed hydrogen bonding with a spacing of 2.1 Å with THR-130; mutant SER-134 formed hydrogen bonding with a spacing of 2.1 Å and 3.5 Å with THR-130, 2.7 Å with LEU-131, 2.4 Å with MET-135, and TYR-138 to form hydrogen bonds with a spacing of 2.0 Å. Mutation of c.146C>T: p.T49M could cause the amino acid threonine to transfer to hydrophobic ones from hydrophilic ones. Mutation of c.287A>G: p.Y96C could cause aromatic-like amino acid changed to sulfur-containing amino acid, which affects the TSPAN12 protein structure: wild-type TYR-96 formed hydrogen bonding with LEU-92 at a spacing of 2.5 Å, LEU-100 at a spacing of 2.2 Å, SER-99 at a spacing of 2.8 Å, and ASN-18 at a spacing of 2.7 Å. The mutant CYS-96 formed hydrogen bonding with LEU-92 at a spacing of 2.2 Å and LEU-100 at a spacing of 2.4 Å. The mutant CYS-96 formed hydrogen bonding with LEU-92 at a spacing of 2.4 Å. The mutant CYS-96 forms a hydrogen bond with LEU-92 at a spacing of 2.2 Å and a hydrogen bond with LEU-100 at a spacing of 2.4 Å (Fig. 6).

## Discussion

Familial exudative vitreoretinopathy (FEVR), as described for the first time in 1969 by Schepens and Criswick, is a hereditary condition which is characterized by peripheral retinal abnormalities and incomplete vascularization [8]. The presence of incomplete and aberrant vascularization is associated with the development of a variety of eye complications, which includes retinal exudates and neovascularization, retinal folds and detachments, vitreous hemorrhage, macular ectopia, and eventually resulting in total blindness.

FEVR has great phenotypic and genetic heterogeneity. Inheritance of FEVR, which is genetically determined, can be dominant, recessive or X-linked, with dominant inheritance being the most common manner. It has been currently found that at least nine genes are identified as responsible for the development of FEVR, which includes NDP, FZD4, LRP5, TSPAN12, ZNF408, KIF11, RCBTB1, CTNNB1, and JAG1 [9]. The proteins from the initial four genes work together in the Norrin/β-catenin signaling pathway, known as the Norrin/Frizzled-4 signaling pathway as well, and exhibit strong mutual interaction [10].

In previous study, we characterized the subtypes of FEVR by genetic analyses combined with clinical symptoms. Significant phenotypic difference was shown in *TSPAN12* mutations with ocular developmental disorder affecting retinal exudates and neovascularization, retinal folds and detachments, vitreous hemorrhage, and macular ectopia [11]. In this study, we investigated a cohort of FEVR patients with *TSPAN12* mutations. Compound heterozygous or homozygous *TSPAN12* variants were found in 6 affected patients with a unique combination of lens and iris abnormalities.

Approximately 47.87% (56 of 117) of the variants were found in 7 genes (*FZD4, LRP5, TSPAN12, NDP, CTNNB1, ZNF408, and KIF11*) among the variants recognized in these study participants. 9 probands were identified as carrying *TPSAN12* mutation, with a mutation detection rate of 15.25%. The highest non-missense mutation proportion was *TSPAN12,* reaching 44.44%.

In this study, these *TSPAN12* variants were identified as being deleterious by virtue of the results of bioinformatic analyses and the rareness in general population, but further functional experimental research were needed to formally confirm their deleterious properties. To understand the clinical significance of *TPSAN12* variants, functional study of the 3 mutations (c.612+1G>A, c.149+2 T>C and c.360G>A) on *TPSAN12* expression was made in this study. Minigene analysis confirmed the 3 mutation on *TPSAN12* found in FEVR patients caused alters splicing resulting in loss of *TPSAN12* expression.

The novel intronic variants in *TPSAN12* was shown a deleterious effect on silico algorithms. An analysis of mini-gene splicing assay in vitro revealed that these variants alter splicing, causing frameshifts and predicted premature termination codons, resulting in protein dysfunction from this allele via nonsense-mediated mRNA decay (NMD).

TSPAN12 is a tetraspanin which is encoded by chromosome 7q31 and composed of 305 amino acids. An intracellular loop and two extracellular loops (ECL-1 and ECL-2) comprise four transmembrane domains. *TSPAN12* was revealed to associate selectively with Norrin/β-catenin pathway as the significant auxiliary constituent part of Norrin/FZD4/LRP5 complex [12]. Through interacting between the FZD4 and LEL, *TPSAN12* is anchored into the Norrin receptor complex*.* It improves its signal transduction and enhances the selectivity of ligand-receptor binding by promoting the polymerization of Norrin/FZD4/LRP5 complex, and ultimately assists in reducing the phosphorylation and degradation of β-catenin, making more β-catenin into the nucleus to act as transcription factors. TSPAN12 participates in retinal angiogenesis significantly under physiological conditions through Norrin/β-catenin signaling pathway. In contrast, *TSPAN12* mutations have been reported to be associated with vascular injury, nerve cell damage, microaneurysms and other retinal vascular diseases [13].

**Fig. 7:**
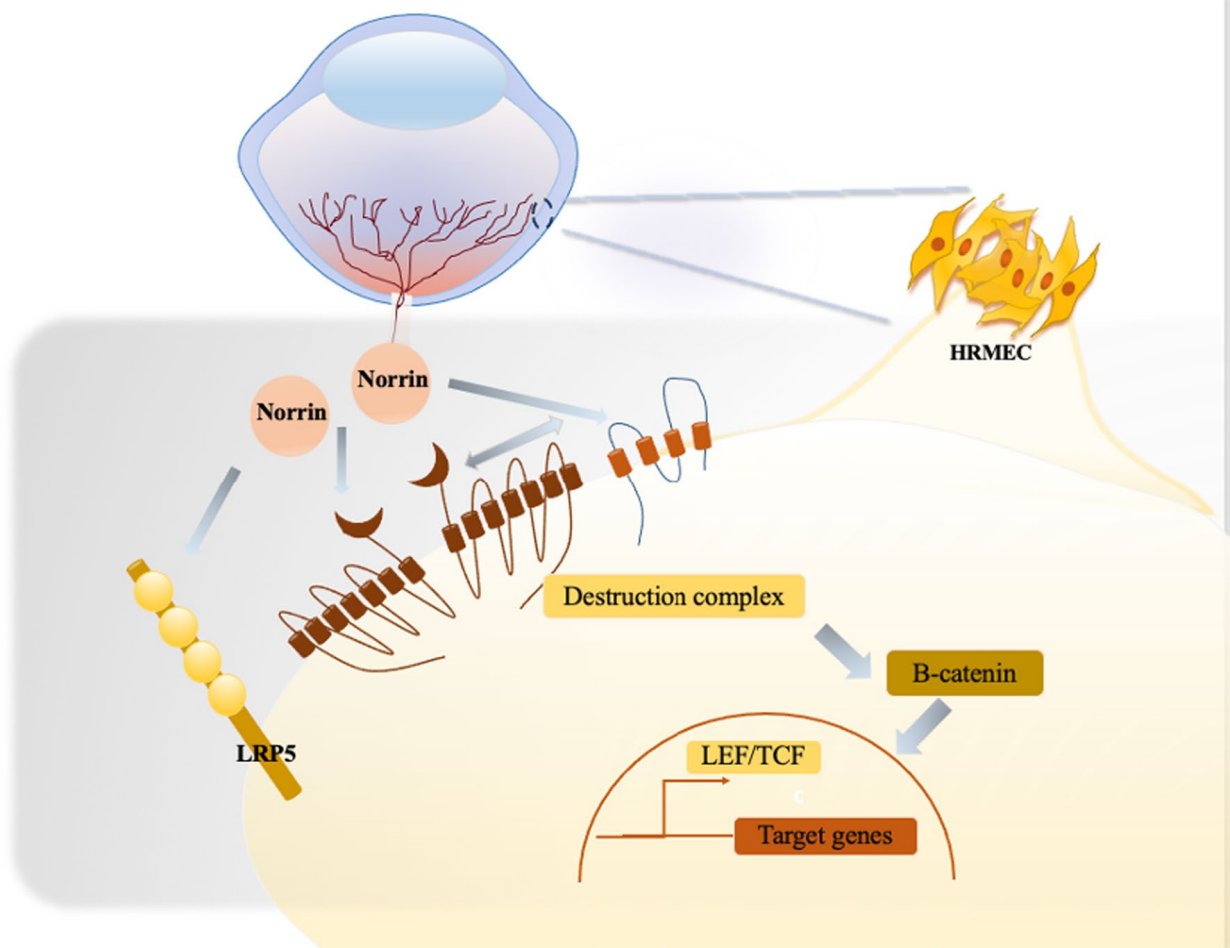
The proposed mechanisms of how *TSPAN12* mutations lead to FEVR

In patients who have complex heterozygous mutations, inactivation of the PTC-containing allele by NMD results in a halving of the total amount of effective TSPAN12 proteins, thus possibly becoming the reason of diseases due to haploinsufficiency. The dominant system between Norrin/β-catenin pathway and Wnt/β-catenin pathway systems presents a difference between numerous regions of the central nervous system, among which the Norrin/β-catenin pathway system makes the major contribution to the retina [14]. Recessive loss-of-function mutations in *TPSAN12* results in aberrant Norrin/β-catenin pathway, rather than affecting Wnt/β-catenin pathway. Therefore, the imbalanced signaling and relative enhancement of the latter become potential risk factors of diseases associated with *TPSAN12* and FEVR. The specific promotion of the Norrin/β-catenin pathway owing to *TSPAN12* mutations will cause spectrum of retinal vascular diseases (Fig. 7).

The detailed molecular mechanism by *TSPAN12* mutations is still lacking. Further integrative functional studies in the comprehension to *TSPAN12* sequence variants is needed. For gaining a deeper understanding of the influence of *TSPAN12* in ocular system, animal model is needed to build in our further study.

## Conclusions

In conclusion, this study expanded the variants spectrum and phenotype of *TSPAN12* gene. Besides, function validation assay showed that the expression of mRNAs and proteins of *TSPAN12* were decreased in transfected cells with mutant *TSPAN12* plasmids. Intronic sequence analysis, increasing the interpretation of molecular mechanism of *TSPAN12* pathogenesis at the transcription and translation levels, might be a vital tool for genetic counseling and prenatal diagnoses.

### Supplementary Information

**Additional file 1**. **Table S1**: The primers uesd in pMINI-gene construction. **Table S2**: The primers uesd in Amplification product identification. **Table S3**: The primers uesd in RT-PCR.

## Data Availability

The datasets used and/or analysed during the current study are available from the corresponding author on reasonable request.

## Abbreviations

FEVR: Familial exudative vitreoretinopathy
NGS: Next-generation sequencing
PTC: Premature termination codons
NMD: Nonsense-mediated mRNA decay
